# Unraveling the Sexual Dimorphism of First Instar Nymphs of the Giant Stick Insect, *Cladomorphus phyllinus* Gray, 1835, from the Atlantic Forest, Brazil

**DOI:** 10.3390/ani13223474

**Published:** 2023-11-10

**Authors:** Jane Costa, Lucas Torres, Leticia Paschoaletto, Ana Luiza Anes Pimenta, Hugo A. Benítez, Manuel J. Suazo, Carolina Reigada, Hélcio R. Gil-Santana

**Affiliations:** 1 Laboratório de Entomologia, Instituto Oswaldo Cruz, Fiocruz, Rio de Janeiro 21040-360, Brazil; luka27.silva@gmail.com (L.T.); alapbio@gmail.com (A.L.A.P.); 2 UFRJ Laboratório de Parasitologia Molecular, Instituto de Biofísica Carlos Chagas Filho, Universidade Federal do Rio de Janeiro, Rio de Janeiro 21941-909, Brazil; paschoaletto@biof.ufrj.br; 3 Centro de Investigación de Estudios Avanzados del Maule, Instituto Milenio Biodiversidad de Ecosistemas Antárticos y Subantárticos (BASE), Universidad Católica del Maule, Talca 3466706, Chile; hbenitez@ucm.cl; 4 Centro de Investigación en Recursos Naturales y Sustentabilidad (CIRENYS), Universidad Bernardo O’Higgins, Avenida Viel 1497, Santiago 8370993, Chile; 5 Instituto de Alta Investigación, CEDENNA, Universidad de Tarapacá, Casilla 7D, Arica 1000000, Chile; suazo.mj@gmail.com; 6 Departamento de Ecologia e Biologia Evolutiva, Universidade Federal de São Carlos, São Paulo 13565-905, Brazil; 7 Laboratório de Diptera, Instituto Oswaldo Cruz, Av. Brasil, 4365, Rio de Janeiro 21040-360, Brazil; helciogil@uol.com.br

**Keywords:** Cladomorphinae, morphology, Neotropical, Phasmida, sexual dimorphism traits

## Abstract

**Simple Summary:**

Members of the order Phasmida are popularly known as walking stick insects. They have remarkable camouflage, resembling moss, sticks, and leaves. *Cladomorphus phyllinus* is a giant stick insect from the Atlantic Forest of Brazil. Described in 1835 by George Robert Gray, the species is widely distributed in the wild, but several aspects of its biology and ecology remain to be studied. An inedited description of the first instar nymphs of *C. phyllinus* is presented, showing clear sexual dimorphism in the thorax and distinct abdominal sternites. A sex-specific suture was identified in the metanotum of males but was absent in females. Differentiation of the last three abdominal segments, which are already shaped to form the copulatory apparatus in both sexes, was also recorded and illustrated. Such sexual dimorphism is rare in first instar insect nymphs. Therefore, this study uncovered new characteristics that allow more precise data generation from samples collected in the field for conducting experiments, recording specimens in entomological collections, and improving the species concept and knowledge of sexual dimorphism in *C. phyllinus*.

**Abstract:**

The first instar nymphs, both male and female, of the giant stick insect *Cladomorphus phyllinus* Gray, 1835 were carefully described and measured, revealing a remarkable sexual dimorphism that is considered rare among insects and is poorly explored in the order Phasmida. The studied F1 nymphs originated in captivity from eggs laid by a coupled female specimen collected in the Atlantic Forest in the vicinity of Petrópolis city, state of Rio de Janeiro, Brazil. The first instar nymphs of *C. phyllinus* were measured and illustrated in high-resolution photographs to show the general aspects and details of sexually dimorphic traits, making clear the phenotypic differences in the sexes. A total of 100 nymphs were kept alive until morphological sexual dimorphism was confirmed and quantified. All recently hatched first instar nymphs were separated based on the presumed male and female characteristics, i.e., the presence and absence of the suture in the metanotum in the males and females, respectively, had their sexes confirmed in 100% of the specimens as previously assigned. These results confirm this new morphological trait, which here is named “alar suture” as sex-specific in the first instar nymphs, a novelty in this stage of development of sexual differentiation. In addition, the distinct conformations of the last three abdominal sternites of both sexes were recorded.

## 1. Introduction

Research on sexual dimorphism (SD) as a source of phenotypic variation in animals is of great interest to ecology and evolutionary biology, probably because the degree of sexual differences in size and shape varies noticeably in different taxa [1,2]. According to Ralls and Mesnick [3], SD can be defined as the differences between the sexes of the same species that are not directly involved in reproduction. Esperk et al. [4] defined SD in size (SSD) as the disparity in body sizes of the two sexes, highlighting that it is a common phenomenon in different groups of animals. The underlying factors contributing to SSD have been subject to thorough investigation and extensive studies have been dedicated to exploring the mechanisms that give rise to SSD [4,5]. In addition, insects exhibit diverse morphological variations in response to their environment, and these variations are often considered adaptive due to their correlation with specific traits (i.e., length of legs, size of elytra, shape of the abdomen, wing size, and differences in shape), which vary across populations [2,6,7].

The Phasmida currently includes more than 3510 species grouped into 531 genera [8,9,10,11]. The group is still little studied, despite the increasing number of researchers working on its distinct aspects [12]. In Brazil, only around 300 species are currently known, and it is anticipated that this number could be significantly higher. Most of the information on this group is original descriptions conducted by naturalists. Madeira-Ott et al. [12] emphasized the articles by Toledo Piza as a significant contribution to the state-of-the-art studies on Brazilian Phasmida. Additionally, several studies on Brazilian taxa in the last 20 years have been conducted on the subject, including taxonomic revisions [13,14,15,16,17,18,19], redescription of iconic taxa such as *Tithonophasma tithonus* (Gray, 1835) and *Pseudophasma cambridgei* Kirby 1904 [20,21], and descriptions of two new species in the genus *Cladomorphus* Gray, 1835 [22,23] and one in *Isagoras* Stål, 1875 [24]. Nevertheless, many aspects of their developmental stages are unknown. The eggs of the Brazilian species, for example, are still very little explored, although their importance in the improvement of the systematics, taxonomic, and phylogenetics is recognized [13,25,26,27,28].

Sexual dimorphism between adults in Phasmida is common, and several species that exhibit fascinating examples of this phenomenon can be cited. Size distinction is one of the most common, although many other morphological features have been described among adults in the order [10]. Some species, however, show a discrete differentiation between sexes; one example is *Paraphasma paulense* Rehn, 1918, which has a tenuous SD, with both sexes exhibiting well-developed hind wings and females being only slightly stouter and longer than males [29]. 

Two new names in the Phasmida order, Oriophasmata (“Eastern phasmids”) and Occidophasmata (“Western phasmids”) were recently proposed based on phylogenetic relationships; the presence or absence of wings in this group has been deeply analyzed not just in terms of SD but also in terms of their evolutionary processes [9]. The use of nuclear genes led to the assumption that the ancestral Phasmatodea was apterous and that wings developed independently several times in subsequent lineages [30]. According to Bank and Bradler [31], who constructed a robust phylogeny encompassing 500 species, this group of insects “provides an expedient study system to explore the evolution of flight due to their high diversity in wing states and sizes varying among closely related species and between sexes”.

The giant stick insect *Cladomorphus phyllinus* can be easily recognized by, among other characteristics, its large size. It is commonly found near cities. However, it is a poorly studied species and is mainly geographically distributed in the Atlantic Forest and other areas in the north and northeast regions of Brazil and Argentina [11,19]. Phenotypical aspects of adults and eggs and some biological features of the mentioned species are already present in the literature [23,32,33,34,35,36]. It has also been used as an interesting species for scientific dissemination and educational training programs for children [37]. 

*Cladomorphus phyllinus* is one remarkable example of SD and SSD since the adults have accentuated phenotypical differences: the males have wings and are smaller and slenderer, reaching only up to 15 cm in length, while the females are apterous, reaching up to 26 cm in length [22,23,38]. However, analysis of SD during the early developmental phase has not been extensively explored in Phasmida; for instance, several decades ago, Leuzinger et al. [39] and Wilbert [40] studied the development of the 8th abdominal sternite in male and female nymphs of *Carausius morosus* Wattenwyl, 1907. Dürr and Mesanovic [41] recently analyzed the development of sexual dimorphism in three stick insect species. Although their study focused on the development of size and body limb proportions, it used characteristics of the 8th abdominal sternite to distinguish male and female 1st instar in *Aretaon asperrimus* (Redtenbacher 1906) and *Medauroidea extradentata* (Wattenwyl 1907).

This study aimed to describe the first instar nymphs of *C. phyllinus* and to analyze and identify patterns of SD, making it the first study to evaluate and characterize these morphological variations in the early developmental stages of *C. phyllinus.*

## 2. Materials and Methods

A colony was started with a coupled female collected in a fragment of the Atlantic Forest in Petrópolis municipality, Rio de Janeiro, Brazil, close to an urbanized site in the city (22°30′18′′ S, 43°10′43′′ W). The sample was collected in accordance with the Brazilian biodiversity policies, under SISBIO license number 12123. The collected specimen was identified as *C. phyllinus* based on the literature and taxonomical keys [10,11,16,22,38], was corroborated using DNA analysis, and compared with previously published studies [12,23].

Two groups of first instar nymphs were studied. Group 1 was composed of 30 live specimens selected and observed in detail under a stereomicroscope. Morphological trait differences were recorded and the nymphs separated based on their similarities and phenotypical traits that might be associated with SD. These specimens were used to describe the first instar features photographed. Later, the specimens were preserved in 80% ethanol and 20 first instar nymphs (10 males and 10 females) were measured (Table 1). Differences between morphological trait measurements of male and female nymphs were compared using the Kruskal–Wallis test since the Shapiro test did not indicate the normality of the data. 

Group 2, which was composed of 100 hatched nymphs that were also selected and separated based on their phenotypical traits, was used to confirm SD. They were kept alive as previously described [23] until they completely developed to the adult stage and then characterized as male or female based on their morphological characteristics, as previously described. 

The nomenclature used in the description of the first instar nymphs was based on Hennemann et al. [16] and Brock and Büscher [10].

Ten voucher specimens of the first instar nymphs of each sex were deposited in the Entomological Collection of the Instituto Oswaldo Cruz.

The stereomicroscope used for the observations was a Zeiss, Stem SV6 (Jena, Germany). Photos of the general aspects of the live specimens, first instar nymphs, and adults were taken using a Samsung Note 8 camera (Samsung Electronics, Suwon, South Korea) with an additional lens. The high-resolution 3D photos were taken in a stereomicroscope LEICA Model M25 C coupled to a camera LEICA DMC 2900 (Wetzlar, Germany) running a Leica Application Suite version 4.7.1 program.

## 3. Results

### 3.1. Phenotypic Description of the First Instar Nymph 

#### 3.1.1. General Aspect

**Figure 1:**
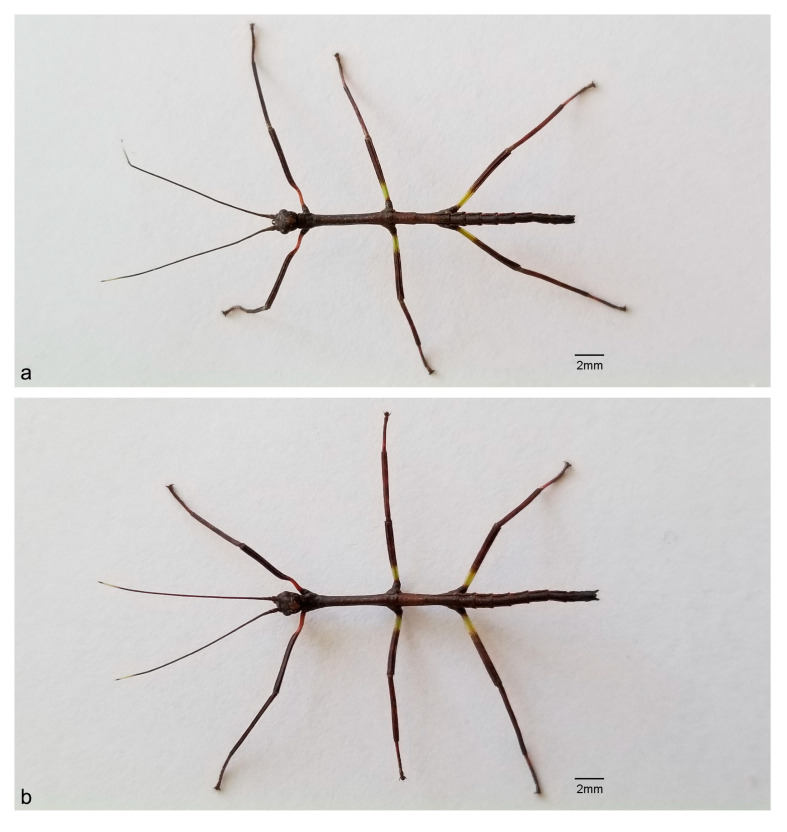
Habitus of *Cladomorphus phyllinus* first instar nymphs. (**a**) male; (**b**) female.

General lengths of males (21.5–22.4 mm) and females (22–22.4 mm) are shown in Figure 1a,b, respectively, and in Table 1.

**Table 1:** Measurements (mm) of first instar *Cladomorphus phyllinus* nymphs showing significant differences between males and females based on overall length, mesothorax length, and anterior and hind legs.

		Mean (±SD)	Median	IQR	$\chi^{2}$	*p*-Value
Overall length	Female	22.2 (±0.151)	22.3	0.175	4.68	<0.05 *
	Male	22.0 (±0.241)	22	0.100		
Head length	Female	1.95 (±0.053)	1.95	0.100	0.192	0.661
	Male	1.96 (±0.0516)	2	0.100		
Head width	Female	2 (±0.067)	2	0	2.887	0.089
	Male	1.95 (±0.053)	1.95	0.100		
Antenna	Female	13.1 (±0.225)	13.0	0.175	0.0247	0.875
	Male	13.1 (±0.212)	13.0	0.200		
Prothorax length	Female	1.18 (±0.103)	1.2	0.175	0	1
	Male	0.092 (±1.2)	1.2	0.075		
Mesothorax length	Female	5.08 (±0.123)	5.05	0.175	4.263	<0.05 *
	Male	4.91 (±0.197)	5	0.075		
Metathorax length	Female	4.28 (±0.162)	4.3	0.175	3.41	0.065
	Male	4.15 (±0.127)	4.1	0.100		
Abdomen length	Female	9.76 (±0.158)	9.8	0.25	0.472	0.492
	Male	9.82 (±0.262)	9.85	0.45		
Fore leg	Female	15.9 (±0.279)	16	0.075	12.351	<0.05 *
	Male	15.2 (±0.179)	15.2	0.275		
Middle leg	Female	13.3 (±0.246)	13.4	0.40	0.997	0.318
	Male	13.2 (±0.211)	13.2	0.425		
Hind leg	Female	16.6 (±0.392)	16.8	0.550	4.229	<0.05 *
	Male	16.2 (±0.217)	16.2	0.350		
Mesothoracic suture	Female	0.99 (±0.110)	1	0.075	2.839	0.092
	Male	1.07 (±0.067)	1	0.100		
Metathoracic suture	Female	1.07 (±0.082)	1.1	0.075	3.344	0.067
	Male	1.13 (±0.048)	1.1	0.075		

* Represents morphological traits with significant differences between males and females (based on the Kruskal–Wallis test).

#### 3.1.2. Coloration

**Figure 2:**
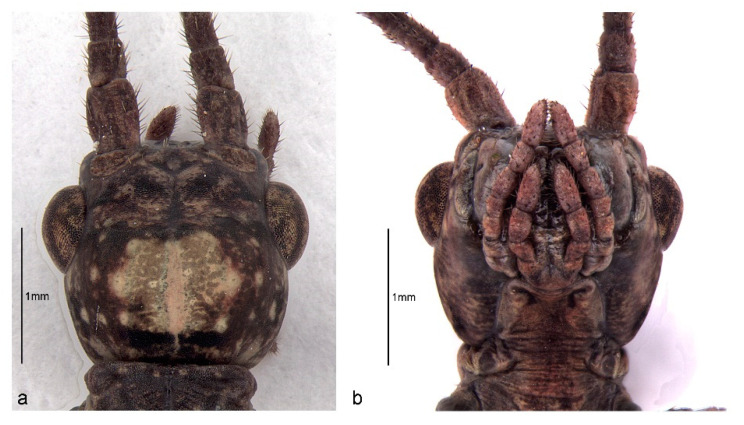
Head of *Cladomorphus phyllinus*: (**a**) dorsal view of a male first instar nymph; (**b**) ventral view of a female first instar nymph.

**Figure 3:**
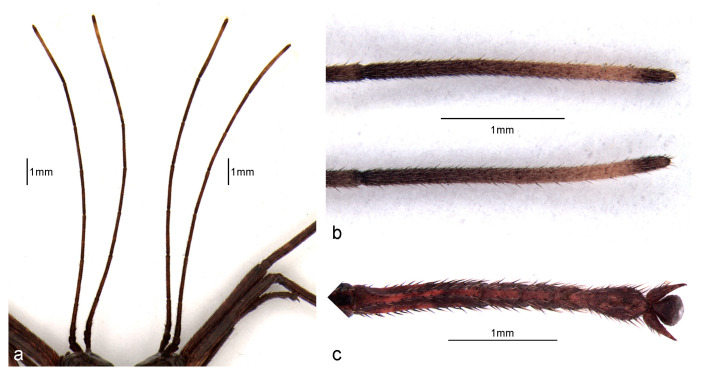
*Cladomorphus phyllinus*. first instar nymph: (**a**) general aspect of the pair of antennae (left: male; right: female); (**b**,**c**) distiflagellomeres and tarsus of the foreleg of a male.

**Figure 4:**
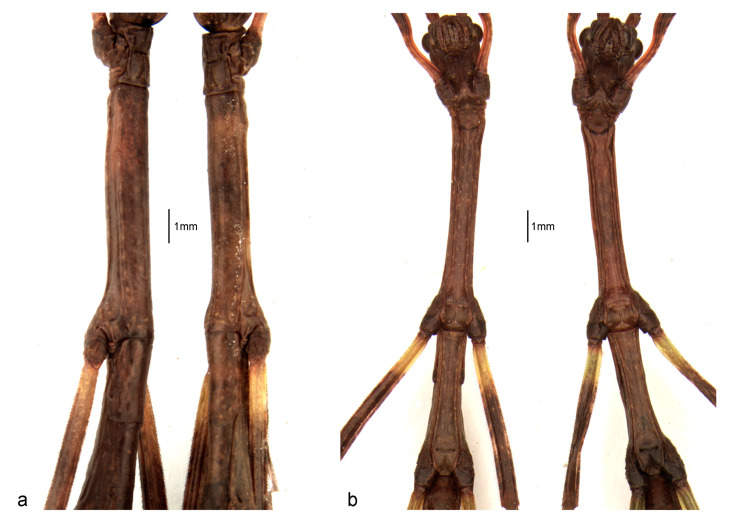
*Cladomorphus phyllinus* (**a**) thorax, lateral view: left is male and right is female; (**b**) idem, ventral view.

**Figure 5:**
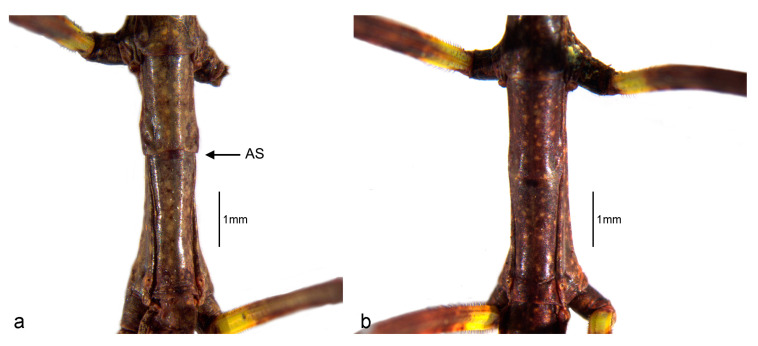
Metanotum of *Cladomorphus phyllinus* first instar nymphs showing the *alar suture* (AS) in the male (**a**) and its absence in the female (**b**).

The body is generally brownish, with small pale brown spots on the head and thorax. The eyes have irregularly intermixed dark and light brown spots. The antennae have distal segments that are paler than the previous ones and darker at their extreme apices. The legs are darker, the basal portion of the anterior femora is reddish brown, and the anterior portions of the median and hind femora are pale yellow–green (Figure 1, Figure 2, Figure 3, Figure 4 and Figure 5).

#### 3.1.3. Structure and Vestiture

The head is globose, integument glabrous, and smooth. The distance between the external margin of the eyes is as long as its length (neck excluded, Table 1). The eyes are rounded in dorsal and lateral view and ocelli are absent. The antennae have nine segments, its apex reaches the second tarsal segment of the fore legs, the scape is flattened dorsoventrally, and the pedicel is cylindrical and half shorter than the scape length. The flagellum has seven segments, which are similar in size and shape, and is homogeneously covered with thin and short setae (Figure 2a,b and Figure 3a,b). Clypeus, labrum, and distal maxillary and labial palpomeres are covered with many tiny setae. The labial palpus has seven segments and the maxillary palpus has four segments, with the segments getting progressively shorter towards the distal end (Figure 2b). All the mentioned characteristics were observed in both males and females.

The thorax is ventral and dorsally convex and the integument is smooth. The prothorax is the shortest thoracic segment and is slightly shorter than the head. The pronotum has a medial longitudinal furrow and a transversal furrow nearer to the anterior margin. The mesothorax is the longest—one-third longer than the metathorax. The males have a transversal suture, here called “alar suture” (AS), which is absent in the females. Wing buds are absent (Figure 4 and Figure 5, Table 1).

**Figure 6:**
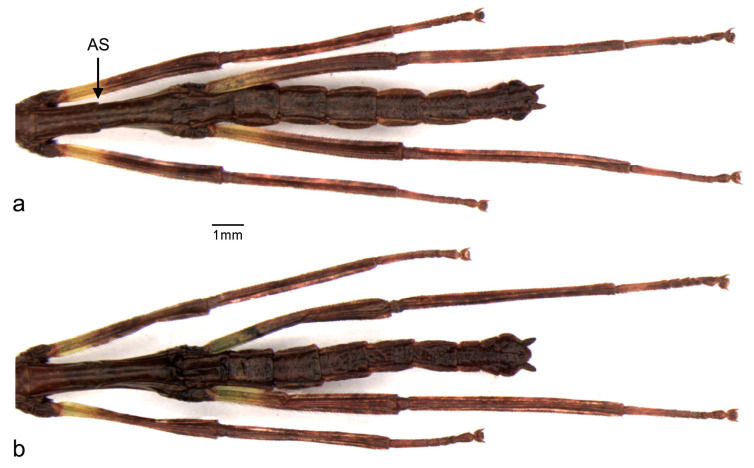
Dorsal view of the metathorax and abdominal segments in first instar nymphs of *Cladomorphus phyllinus*: (**a**) male, (**b**) female.

Cursorial legs, coxae, and trochanters are almost glabrous. The femora and tibiae have short and thin setae, which form rows. The hind legs are the longest. The tarsi pentamerous are homogeneously covered by thin and small setae and the first segment is longer than the others. The arolium is well-developed, gibbous, and slightly shorter than the tarsal claws (Figure 1, Figure 3 and Figure 6).

**Figure 7:**
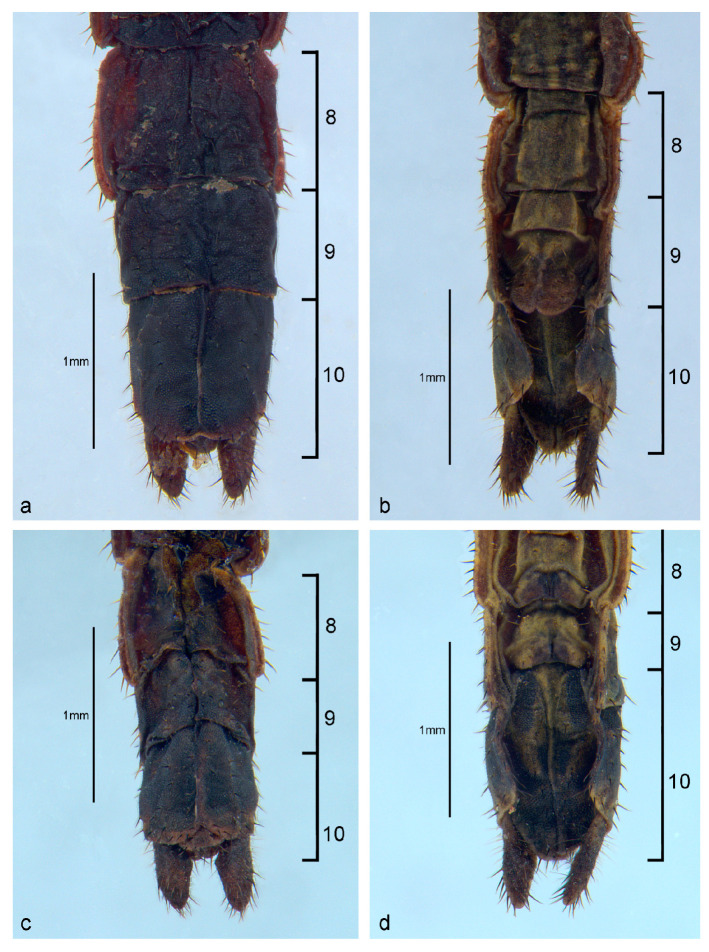
Last three abdominal segments (8,9,10) in the first instar nymphs of *Cladomorphus phyllinus*: (**a**,**b**) male, (**c**,**d**) female, (**a**,**c**) dorsal view, (**b**,**d**) ventral view.

The abdomen has 10 visible segments (the 1st one, the median, is fused to the metathorax and is not clearly defined in all specimens). There are long rows of sparse and short setae on lateral portions; tergite IX is the shortest. There is one pair of small cerci at the apex that is smaller than tergite X and is covered by setae. The shapes of the last three sternites and their respective differentiation based on sex are marked in the ventral view (Figure 6). In males, sternite VIII has a straight posterior border and sternite IX has a bifurcated center and curved lobes. In females, sternite VIII has a suture near its posterior border, while sternite IX has a median suture with a straight border (Figure 6 and Figure 7). 

### 3.2. Sexual Dimorphism in First Instar Nymphs

SD in first instar nymphs was observed in terms of both size and morphology. In morphology, SD presents as a bimodular configuration and is evident in the metanotum and the last three abdominal sternites. Males have a clear suture slightly anterior to the middle line of the metanotum (AS). Here, the AS was recorded and named for the very first time as a new sex-specific character. This suture is absent in females. The conformation of the sternites in the last three abdominal segments is also distinct in male and female first instar nymphs (Figure 5, Figure 6 and Figure 7).

SSD was also detected by measuring morphological traits, including 13 variables, four of which (general length, mesonotum, and fore and hind legs) showed statistically significant differences. However, the four morphological traits that were different between sexes had similar values; therefore, SD in first instar nymphs of *C. phyllinus* is more clearly expressed by morphologically conspicuous traits (AS and differentiation of the last three abdominal segments) than by SSD.

A total of 100 specimens were followed up from hatching to confirmation of their sex based on the presence or absence of the AS. There were 47 male and 53 female specimens, which corresponded with the previously presumed sexes. Their development was followed up until the adult stage: seven and nine moults were recorded in males and females, respectively.

**Figure 8:**
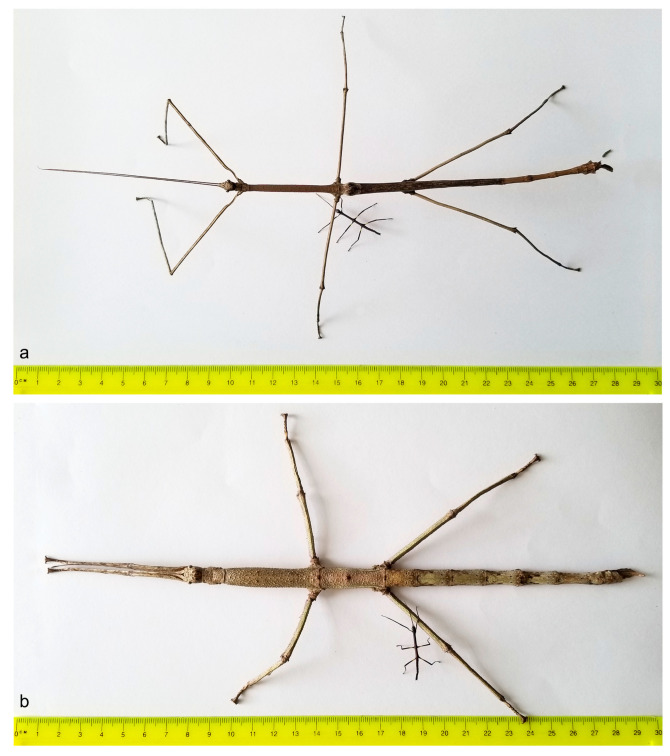
Remarkable size differences between *Cladomorphus phyllinus* first instar nymphs and adults. (**a**) male and (**b**) female.

Despite the evident morphological distinctions associated with SD in first instar nymphs of male and female *C. phyllinus*, SD in this early instar is slight compared with adults. While the difference in lengths between sexes is an average of 1.34% in first instar nymphs, it reaches 42% in adults. The lengths of female first instar nymphs increased approximately 13 times, while the lengths of male first instar nymphs increased 8 times by adulthood (Figure 1 and Figure 8).

## 4. Discussion

This study allowed us to better understand that SD and processes of sex differentiation in *C. phyllinus* occur early in its developmental stage and are noticeable at the beginning of its metamorphosis. Our results on the morphology of first instar nymphs of *C. phyllinus* broaden the specific concept since first instar nymphs are described for the first time and the remarkable morphological differentiation in the recently hatched nymphs show SD both in the thorax and the last three abdominal sternites. The genital structures of most phasmid species still need to be analyzed [10]. More detailed studies are needed to better characterize the incipient structures in the last sternites of *C. phyllinus* following genital development from initial formation—mainly the gonapophyses and gonoplacs in the females and the poculum and vomer in the males—to more evolved instars [10,16]. It is important to mention that AS, described here for the first time, is directly associated with alar formation. In this experiment, it was followed up until adulthood. A detailed characterization of its development throughout the biological cycle in male *C. phyllinus* is in progress. This characteristic is directly linked to the male strategy of moving/flying in search of females to copulate with, as mentioned in several studies in the literature. In contrast to the males, the females are adapted to producing large numbers of eggs, have a sedentary behavior, and are less exposed to predators [10].

In hemimetabola, being the case for Phasmida, the insects’ developmental pattern goes through gradual morphological changes and the nymphal instars are morphologically similar to the adults, while sexual differentiation, in general, appears in the latest nymphal instars [42]. However, as already mentioned, studies on SD are mainly focused on the adult phase and the latest instars, while the juveniles are often ignored [43,44]. In Phasmida, SD in early instar was previously observed by Leuzinger et al. [39], who revealed the development of the operculum of the 8th abdominal sternite and the visibility of the genital valves in stages 1–6 of female *C. morosus*, while Wilbert [40] compared female, male, and 1st and 2nd instar nymphs of the same species. The first study showed the development of the operculum of the 8th abdominal sternite and the visibility of the genital valves from the first to the sixth instars of *C. morosus*; the second study compared both sexes and the 2nd instar nymphs of the same species.

Early SD and other related traits in insects have been studied in a few groups of Hemiptera, including hematophagous heteropteran bugs (bed bugs, Cimicidae) [45], Chagas disease vectors (Reduviidae, Triatominae) [46,47], and in phytophagous species of the genus *Apiomorpha* Rübsaamen, 1894 (Hemiptera, Coccoidea, and Eriococcidae). In the latter, sexual dimorphism is linked to variations in dispersal and preference for settling sites between the sexes during the crawler stage. In the *A. pharetrata* and *A. munita* species groups, first instar males exclusively choose to settle on the galls that are produced by their mothers. In the case of *A. munita*, males may also opt for galls produced by nearby females [48]. SD of the first instar nymphs of *C. phyllinus* represented by the AS is correlated with dispersal adaptations since it leads to the development of wings in adult males, as observed in the present study. 

Despite the morphological SD and SSD of the *C. phyllinus* adults [10,22,23,38], no size differences were observed in the first instar nymphs. This is similar to the analysis conducted by Esperk et al. [4] with respect to the achievement of high SSD in insects, which occurs gradually through additional instars and longer sexual maturation time, indicating that the additional instars may differ notably between sexes. According to previous studies on the bionomy of *C. phyllinus* [32,33], the males had 5–6 instars over about 100 days, while the females had 9–10 instars over approximately 180 days. Therefore, the remarkable distinction in evolutionary developmental strategies reported between the sexes of the species in relation to the number of ecdysis and developmental time, the marked morphological SD, and the lack or slight differences in the size of the first instar nymphs observed in this study are concurrent with the analyses conducted by Esperk et al. [4].

According to Dürr and Mesanovic [41] “in addition to the strong inter-specific variation of body: limb proportions, stick insects also show considerable intra-specific variation both among developmental stages and between sexes. For example, adult male stick insects are generally smaller and slenderer than their female conspecifics, also bearing relatively longer legs and antennae. Intriguingly, strong sex-specific differences in overall body size and shape are not apparent in young larvae”. Despite the significant statistical differences observed in the four variables measured in first instar nymphs of *C. phyllinus*, the values obtained for males and females were very close, therefore SSD is not as clearly expressed as the recorded morphological traits (AS and the last three abdominal segments). Nevertheless, *C. phyllinus* adults have remarkable SD and SSD [22,23,38]. 

In phasmids, most of the species are apterous and there is a high diversity in wing state, morphology, and size among closely related species, as well as between sexes [31,49,50]. As an example of this variation in wings, we mention the spiny stick insect *Extatosoma tiaratum* (Macleaty 1827), a well-studied and sexually dimorphic phasmid that is endemic to tropical rainforests in north-eastern Australia [51]. The females are brachypterous while the males’ wings are well-developed and functional [52]. The other example is *P. paulense*, a Brazilian species from the Atlantic Forest, in which both sexes have well-developed wings [29]. Its first instar nymph was described, and no information was found on the AS in the thorax or on any SD trait. Whether AS can be identified in both sexes of first instar nymphs of both species is still to be explored. As previously reported, the majority of studies on the morphological, behavioral, and ecological traits of the phasmids have focused mainly on adults; therefore, no references on this topic were found for further discussions and comparisons with the *C. phyllinus* AS. Our study showed that AS is a new trait associated with SD. As observed for *C. phyllinus*, AS enabled the separation, without errors, of the males and females in recently hatched first instar nymphs. Further analyses are in progress to reveal and characterize AS development in *C. phyllinus* and in other winged and brachypterous species.

Interestingly, according to a recent phylogenetic study [9], the origin of Cladomorphinae, a subfamily distributed in the Americas and in which *C. phyllinus* is included, would have been in the Asian region. This finding can be interpreted as “the result of a transoceanic, most probably trans-Pacific dispersal event, or via Antarctica which was connected to Australia and South America” and highlights the importance of a better understanding of the evolutionary processes of this group of insects. In addition, studies of the diversity of the Phasmida are mostly focused on the description of new species, while studies that analyze the species in terms of biology, ecology, and physiology are scarce [10]. *Cladomorphus phyllinus* is one of the most poorly studied species in this group of insects in Brazil [23,32,33,34,35] and the morphological aspects of the first instar nymphs have not been analyzed and investigated in detail until now. For the first and second instar nymphs, sexual dimorphism is rare and has been little explored in Phasmida. A great number of Phasmida species have an accentuated SD [10] and it is therefore possible that the expression of SD in early instar nymphs may have remained unrecorded thus far and may be revealed to be more common than is currently known. 

## 5. Conclusions

The results presented here not only broaden the specific concept of the giant stick insect of the Atlantic Forest, since the revised and novel description of the first instar nymphs is presented but also gives more precise data on field captures and samples deposited in entomological collections. Description of first instar nymphs based on stable coloration and comprehensive analysis of morphological traits enabled the definition of SD based on a reliable marker: the presence of a transversal suture in the metanotum of male first instar nymphs, which is absent in females. This suture, named *alar suture* (AS), represents a new sex-specific character in first instar nymphs and a novelty in the morphology of Phasmida. Additionally, the last three abdominal sternites are morphologically distinct in the sexes at this stage of development and are considered a rare event in insects. Measurements of the first instar nymphs showed that size differences are less accurate for sex identification than a conspicuous morphological character such as AS. It is likely that there are many other undiscovered cases of sexual dimorphisms in early instars in Phasmida; therefore, analysis of the morphology of sexually dimorphic species is the first step in addressing other relevant questions on bionomy, evolutionary processes, and ecology, including understanding the sex ratio, which is now in progress. These results open new avenues for further approaches and enlightenment of the evolutionary process of sexual dimorphism in phasmids.

## Data Availability

All relevant material is presented in the manuscript and specimens can be accessed at the Entomological Collection of the Instituto Oswaldo Cruz.
